# Fentanyl but Not Morphine or Buprenorphine Improves the Severity of Necrotizing Acute Pancreatitis in Rats

**DOI:** 10.3390/ijms23031192

**Published:** 2022-01-21

**Authors:** Emese Réka Bálint, Gabriella Fűr, Balázs Kui, Zsolt Balla, Eszter Sára Kormányos, Erik Márk Orján, Brigitta Tóth, Gyöngyi Horváth, Edina Szűcs, Sándor Benyhe, Eszter Ducza, Petra Pallagi, József Maléth, Viktória Venglovecz, Péter Hegyi, Lóránd Kiss, Zoltán Rakonczay

**Affiliations:** 1 Department of Pathophysiology, University of Szeged, 6725 Szeged, Hungary; bioemese@gmail.com (E.R.B.); gabriella.fur@gmail.com (G.F.); ballatanar@gmail.com (Z.B.); kormanyoseszter@gmail.com (E.S.K.); eorjan@gmail.com (E.M.O.); tothxbrigi@gmail.com (B.T.); 2 Department of Medicine, University of Szeged, 6725 Szeged, Hungary; k.kubali@gmail.com (B.K.); pallagi.petra@gmail.com (P.P.); jozsefmaleth1@gmail.com (J.M.); hegyi2009@gmail.com (P.H.); 3 Department of Physiology, University of Szeged, 6725 Szeged, Hungary; horvath.gyongyi@med.u-szeged.hu; 4 Institute of Biochemistry, Biological Research Center, 6726 Szeged, Hungary; szucs.edina@brc.hu (E.S.); benyhe.sandor@brc.hu (S.B.); 5 Department of Pharmacodynamics and Biopharmacy, Faculty of Pharmacy, University of Szeged, 6725 Szeged, Hungary; ducza@pharm.u-szeged.hu; 6 Department of Pharmacology and Pharmacotherapy, University of Szeged, 6725 Szeged, Hungary; vviki3@gmail.com; 7 Institute for Translational Medicine, Medical School, University of Pecs, 7624 Pecs, Hungary

**Keywords:** acute pancreatitis, fentanyl, morphine, buprenorphine, opioids, analgesia

## Abstract

Opioids are widely used for the pain management of acute pancreatitis (AP), but their impact on disease progression is unclear. Therefore, our aim was to study the effects of clinically relevant opioids on the severity of experimental AP. Various doses of fentanyl, morphine, or buprenorphine were administered as pre- and/or post-treatments in rats. Necrotizing AP was induced by the intraperitoneal injection of L-ornithine-HCl or intra-ductal injection of Na-taurocholate, while intraperitoneal caerulein administration caused edematous AP. Disease severity was determined by laboratory and histological measurements. Mu opioid receptor (MOR) expression and function was assessed in control and AP animals. MOR was expressed in both the pancreas and brain. The pancreatic expression and function of MOR were reduced in AP. Fentanyl post-treatment reduced necrotizing AP severity, whereas pre-treatment exacerbated it. Fentanyl did not affect the outcome of edematous AP. Morphine decreased vacuolization in edematous AP, while buprenorphine pre-treatment increased pancreatic edema during AP. The overall effects of morphine on disease severity were negligible. In conclusion, the type, dosing, administration route, and timing of opioid treatment can influence the effects of opioids on AP severity. Fentanyl post-treatment proved to be beneficial in AP. Clinical studies are needed to determine which opioids are best in AP.

## 1. Introduction

Acute pancreatitis (AP) is one of the most common causes for hospitalization within gastrointestinal diseases [1], which has an overall mortality of about 2% [2]. This death proportion in severe cases can increase to 30%. The incidence of the disease is more than 30 per 100,000 population in Europe, and this number has increased over time [3,4]. Excessive alcohol consumption and gallstone diseases account for approximately 70% of cases [2,5]. AP can present in mild, moderately severe, and severe forms based on the Revised Atlanta Classification [6]. The pathomechanism of AP is rather complex, and our understanding of the disease is far from complete, but it involves toxic cellular Ca^2+^ overload causing NF-κB activation, impaired autophagy, mitochondrial dysfunction, and the early intra-acinar and intra-ductal activation of digestive enzymes [7,8,9,10]. The clinical symptoms of AP include severe abdominal pain (which can radiate to the back), fever, nausea, and vomiting. The diagnostic criteria for AP include the presentation at least two of the following: (i) upper abdominal pain, (ii) >3× elevated serum amylase or lipase, and/or (iii) imaging (CT, MRI, ultrasonography) [6,11]. Notably, pain is present in 95% of AP patients [5]. The therapy of AP is only supportive, and there is no specific drug against this disease. Recent AP management guidelines highlight the importance of (a) early intravenous (i.v.) fluid resuscitation; (b) analgesics; (c) enteral nutrition [12,13,14,15]. 

As pain is the most prominent symptom of AP, its relief is a priority in clinical settings. Unfortunately, recent guidelines for AP treatment do not have clear recommendations for the types of analgesics to be used [12,13,16]. Most commonly, the WHO pain management guideline is utilized, and treatment ranges from nonsteroidal anti-inflammatory drugs (NSAID) to high potent opioids. The latter are applied in cases of severe AP and include fentanyl (FE), buprenorphine (BQ), pethidine, pentazocine, morphine (MO), etc. [17]. Although opioids are the most effective pain killers, which makes them valuable in clinical settings, there is a scientific debate on their use due to their side effects such as constipation or immunosuppression [18,19]. Actually, Meng et al. (2013) attempted to collect all randomized controlled trials that investigated the side effects of analgesics (opioids and non-opioids) in AP, but the included studies were of low quality, without clear outcome. However, the use of MO is often not preferred in humans due to the spasm of sphincter of Oddi, which might worsen the outcome of AP [20]. Even more importantly, Barlass et al. [21] have also shown the drawbacks of MO use in AP and the pathological processes of its side effects in a mouse model.

Despite the dubious benefits of opioid use, their impact on the progression of AP is unclear. Therefore, our aim was to investigate opioid receptor function, the effects of FE, MO, and BQ on the severity of AP in rats. We utilized different AP models with opioid pre- and/or post-treatments.

## 2. Results

### 2.1. The Effect of Fentanyl Pre-Treatment on AP Severity

**Figure 1:**
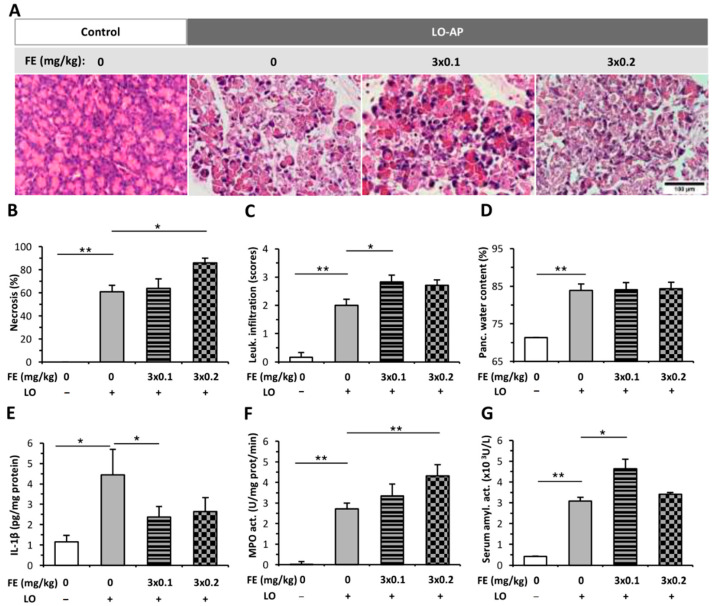
Fentanyl (FE) pre-treatment in L-ornithine (LO)-induced necrotizing acute pancreatitis (AP). Rats were treated with 3 × 0.1 or 3 × 0.2 mg/kg FE intraperitoneally (i.p.), whereas i.p. injection with 3 g/kg LO-HCl (LO +) was used to induce AP. Control animals received physiological saline instead of LO (LO −) or FE (0 mg/kg). Animals were sacrificed at 24 h after LO or physiological saline injection. (**A**) Representative histopathological images of pancreatic tissues of the treatment groups. Bar charts show the extent of pancreatic (**B**) necrosis, (**C**) leukocyte infiltration, (**D**) water content, (**E**) interleukin-1β (IL-1β) concentration, (**F**) myeloperoxidase (MPO) activity, and (**G**) serum amylase activity measurements. Values represent means with standard error, *n* = 9–11. Two-way ANOVA was performed followed by the Holm–Sidak post hoc test. * *p* < 0.05; ** *p* < 0.001.

The pancreata of the control group displayed normal morphology (Figure 1A), and intraperitoneal (i.p.) FE alone did not induce any structural changes in the pancreas (Figure S1A). L-ornithine (LO)-induced AP resulted in about 60% pancreatic necrosis and intensive leukocyte infiltration (Figure 1A–C). These signs even worsened due to FE pre-treatment. The extent of tissue necrosis significantly increased when the higher dose (3 × 0.2 mg/kg) of FE was applied, whereas the level of leukocyte infiltration was higher in the 3 × 0.1 mg/kg FE and AP group compared to the AP group not receiving FE. FE treatment did not cause any change in pancreatic water content in the AP groups (Figure 1D). Serum amylase activity markedly increased in the AP groups versus the control group (Figure 1G). Importantly, 3 × 0.1 mg/kg FE significantly increased serum amylase activity during AP. MPO activity was greatly elevated in the AP groups compared to the control group (Figure 1F), and the dose of 3 × 0.2 mg/kg FE further increased MPO activity in AP. Interestingly, the concentration of pancreatic IL-1β significantly decreased due to 3 × 0.1 mg/kg FE in the AP group.

**Figure 2:**
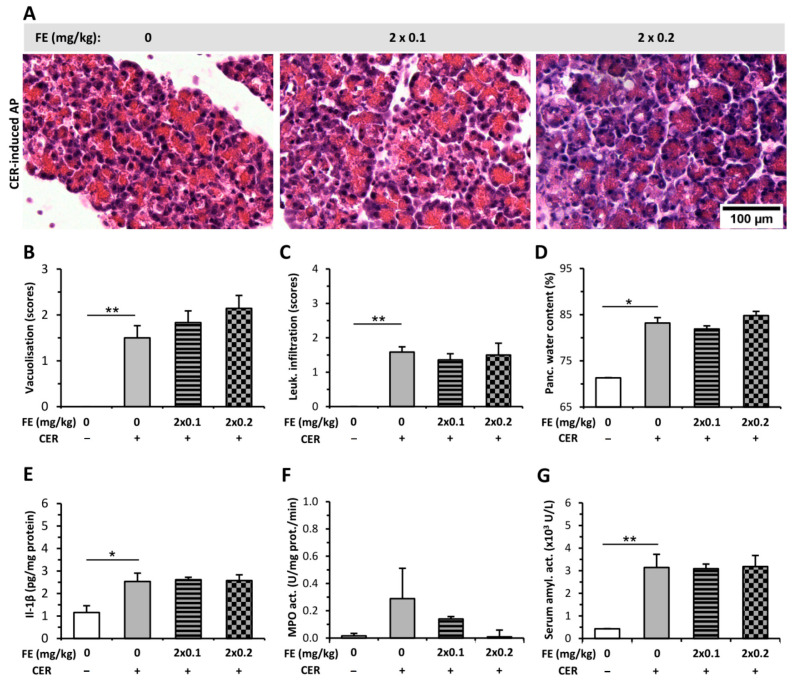
Fentanyl (FE) treatment started before the induction of mild acute pancreatitis (AP) with cerulein (CER) does not affect disease severity. Rats were treated with 2 × 0.1 or 2 × 0.2 mg/kg FE i.p., whereas i.p. injection with 4 × 20 µg/kg CER (CER +) was used to induce AP. Control animals received physiological saline instead of CER (CER −) or FE (0 mg/kg). Animals were sacrificed at 12 h after the first CER or physiological saline injection. (**A**) Representative histopathological images of pancreatic tissues of the treatment groups. Bar charts show the extent of pancreatic (**B**) vacuolization, (**C**) leukocyte infiltration, (**D**) water content, (**E**) interleukin-1β (IL-1β) concentration, (**F**) myeloperoxidase (MPO) activity, and (**G**) serum amylase activity measurements. Values represent means with standard error, *n* = 5–7. Two-way ANOVA was performed followed by the Holm–Sidak post hoc test. * *p* < 0.05; ** *p* < 0.001.

I.p. injections of CER induced mild AP and increased the extent of pancreatic vacuolization, leukocyte infiltration, and water content (Figure 2A–D) compared to the control group (histology of control is shown in Figure S1). FE pre-treatment did not cause any change during AP progression in histological parameters or water content (Figure 2A–D). CER-induced AP resulted in elevated pancreatic IL-1β content and serum amylase activity, whereas it did not significantly affect MPO activity (Figure 2E–G). FE pre-treatment did not alter IL-1β level, MPO, or serum amylase activity in the AP groups.

### 2.2. The Effect of Fentanyl Post-Treatment on AP 

**Figure 3:**
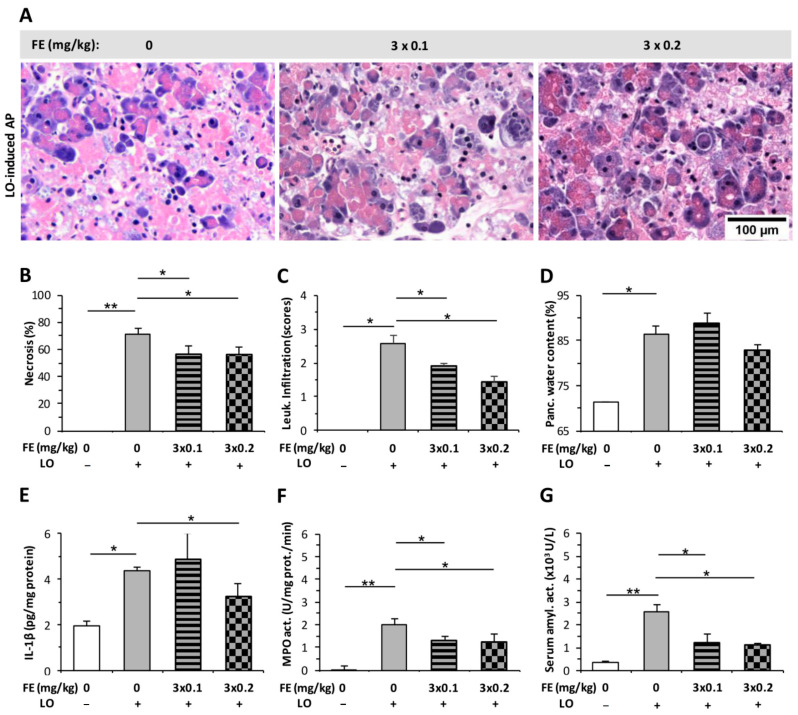
Fentanyl (FE) treatment started after the induction of L-ornithine (LO) acute pancreatitis (AP) reduces disease severity. (**A**) Representative histopathological images of pancreatic tissues of the treatment groups. Bar charts show the extent of pancreatic (**B**) necrosis, (**C**) leukocyte infiltration, (**D**) water content, (**E**) interleukin-1β (IL-1β) concentration, (**F**) myeloperoxidase (MPO) activity, and (**G**) serum amylase activity measurements. Values represent mean with standard error, *n* = 10–18. Two-way ANOVA was performed followed by the Holm–Sidak post hoc test. * *p* < 0.05; ** *p* < 0.001.

In contrast to FE pre-treatment (Figure 1), both doses of FE post-treatment decreased the extent of histopathological changes (pancreatic tissue necrosis and leukocyte infiltration) caused by LO-induced AP (Figure 3A–C). On the other hand, FE administration did not alter pancreatic water content in the AP groups (Figure 3D). LO-induced AP increased pancreatic MPO and serum amylase activities, which were decreased by both FE doses tested (Figure 3F,G). Pancreatic IL-1β levels only decreased significantly in case of the LO + 3 × 0.2 mg/kg FE group (Figure 3E).

**Figure 4:**
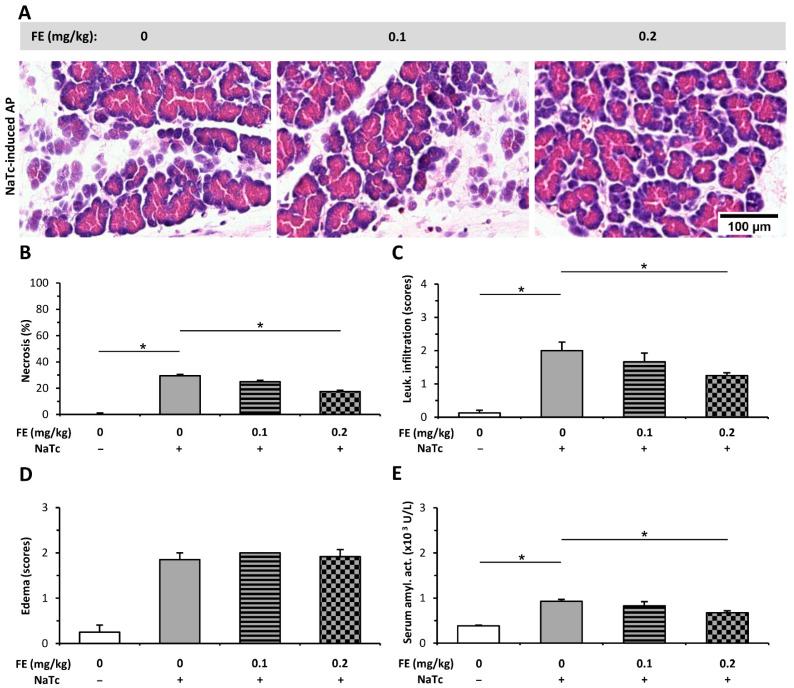
Fentanyl (FE) treatment started after the induction of necrotizing acute pancreatitis (AP) with sodium taurocholate (NaTc) reduces disease severity. Rats were treated with 0.1 or 0.2 mg/kg FE i.p., whereas the intra-ductal injection of 40 mg/kg NaTc (NaTc +) was used to induce AP. Control animals received physiological saline instead of NaTc (NaTc −) or FE (0 mg/kg). Animals were sacrificed at 16–24 h after the NaTc or physiological saline injection. (**A**) Representative histopathological images of pancreatic tissues of the treatment groups. Bar charts show the extent of pancreatic (**B**) necrosis, (**C**) leukocyte infiltration, (**D**) edema, and (**E**) serum amylase activity measurements. Values represent mean with standard error, *n* = 9–12. Two-way ANOVA was performed followed by the Holm–Sidak post hoc test. * *p* < 0.05.

Intra-ductal (i.d.) infusion of sodium taurocholate (NaTc) induced necrotizing AP in the head but not in the tail of the pancreas (not shown), which is in accord with the finding of others [22]. Therefore, only the pancreatic heads were used for analysis. NaTc also elevated the extent of pancreatic necrosis, leukocyte infiltration, and edema (Figure 4A–D). Both necrosis and immune cell infiltration were decreased by the higher dose of FE (0.2 mg/kg, Figure 4B,C), while the score of edema did not change in the AP groups after FE treatment (Figure 4D). Serum amylase activity also decreased in the NaTc + 3 × 0.2 mg/kg FE group versus the AP group without FE treatment (Figure 4E).

**Figure 5:**
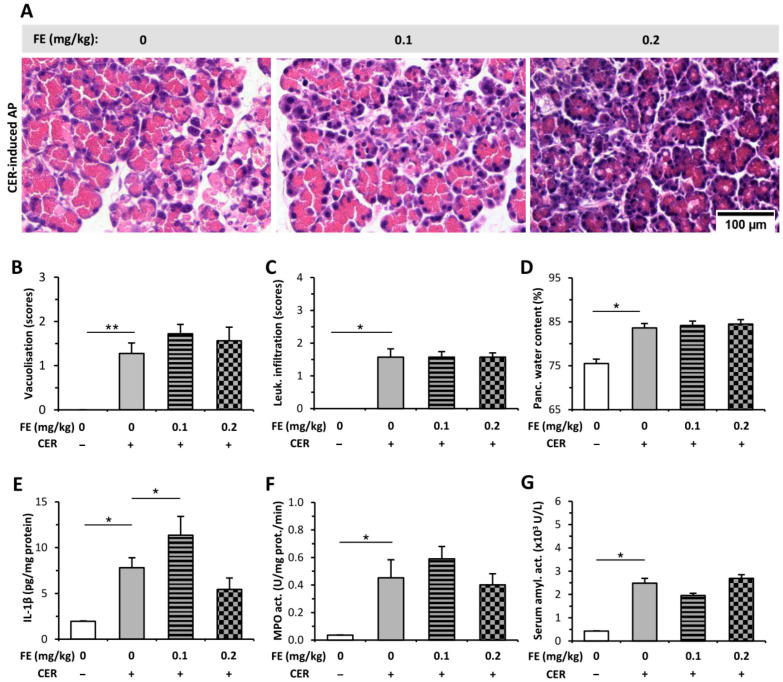
Fentanyl (FE) treatment started after the induction of acute pancreatitis (AP) with cerulein (CER) does not affect disease severity. (**A**) Representative histopathological images of pancreatic tissues of the treatment groups. Bar charts show the extent of pancreatic (**B**) vacuolization, (**C**) leukocyte infiltration, (**D**) water content, (**E**) interleukin-1β (IL-1β) concentration, (**F**) myeloperoxidase (MPO) activity, and (**G**) serum amylase activity measurements. Values represent mean with standard error, *n* = 6. Two-way ANOVA was performed followed by the Holm–Sidak post hoc test. * *p* < 0.05; ** *p* < 0.001.

I.p. injections of CER increased the extent of pancreatic vacuolization, leukocyte infiltration, and tissue water content causing mild edematous AP (Figure 5A–D). FE treatment did not affect either histological parameters (tissue necrosis, leukocyte infiltration) or pancreatic water content (Figure 5A–D). The elevated amylase and MPO activities during AP were unaffected by FE post-treatment (Figure 5E,F). Interestingly, the smaller dose of FE (0.1 mg/kg) further increased the elevated serum IL-1β during AP, while the higher dose of FE had no effect (Figure 5G). 

### 2.3. Morphine Administration Does Not Affect the Severity of AP

**Figure 6:**
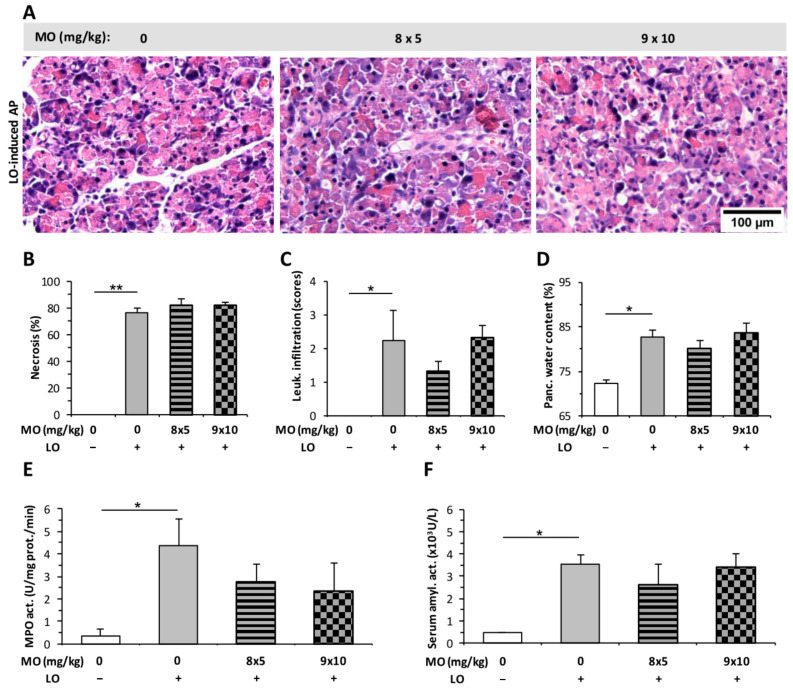
Morphine (MO) treatment does not affect the severity of L-ornithine (LO)-induced acute pancreatitis (AP). Rats were treated with 8 × 5 or 9 × 10 mg/kg MO i.p., whereas 3 g/kg LO-HCl (LO +) was used i.p. to induce AP. Control animals received physiological saline instead of LO (LO −) or MO (0 mg/kg). Animals were sacrificed at 24 h after LO or physiological saline injection. (**A**) Representative histopathological images of pancreatic tissues of the treatment groups. Bar charts show the extent of pancreatic (**B**) necrosis, (**C**) leukocyte infiltration, (**D**) water content, (**E**) myeloperoxidase (MPO) activity, and (**F**) serum amylase activity measurements. Values represent mean with standard error, *n* = 6. Two-way ANOVA was performed followed by the Holm–Sidak post hoc test. * *p* < 0.05; ** *p* < 0.001.

The effect of MO on the severity of AP was investigated by using different doses of the drug: 8 × 5, 9 × 10, and 4 × 5 mg/kg. MO at the tested doses did not induce any structural changes in the pancreatic tissues of rats, and no inflammatory cell infiltration could be observed in histological sections (Figure S1B). Treatment with LO induced AP and resulted in marked pancreatic damage (tissue necrosis, leukocyte infiltration, and increased pancreatic water content Figure 6A–D). MO did not significantly alter the value of these parameters during AP. Furthermore, MO did not influence pancreatic MPO or serum amylase activity in the AP groups, either (Figure 6E,F).

**Figure 7:**
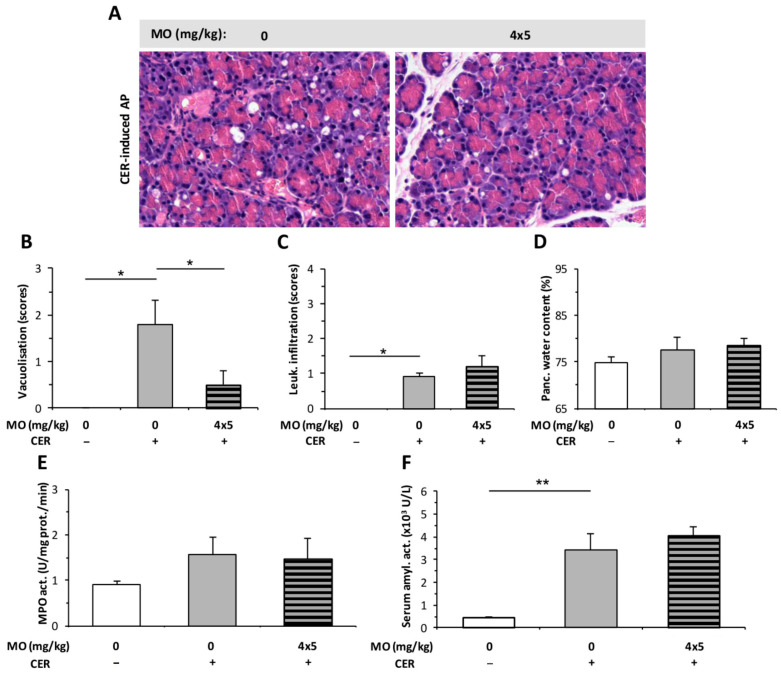
Morphine (MO) treatment does not affect the severity of cerulein (CER)-induced acute pancreatitis (AP). Rats were treated with 4 × 5 mg/kg MO i.p., whereas 4 × 20 µg/kg CER (CER +) was used i.p. to induce AP. Control animals received physiological saline instead of CER (CER −) or MO (0 mg/kg). Animals were sacrificed at 12 h after the first CER or physiological saline injection. (**A**) Representative histopathological images of pancreatic tissues of the treatment groups. Bar charts show the extent of pancreatic (**B**) vacuolization, (**C**) leukocyte infiltration, (**D**) water content, (**E**) myeloperoxidase (MPO) activity, and (**F**) serum amylase activity measurements. Values represent mean with standard error, *n* = 6. Two-way ANOVA was performed followed by the Holm–Sidak post hoc test. * *p* < 0.05; ** *p* < 0.001. Scale bar.

The effect of 4 × 5 mg/kg MO was tested in a CER-induced AP model. Due to the shorter duration of AP in case of CER (12 h) compared to the LO model (24 h), the number of MO injections was reduced from eight (applied in LO-induced AP, Figure 6) to four. In the CER-induced edematous AP, MO significantly reduced vacuolization (Figure 7A,B), but it had no effect on leukocyte infiltration or pancreatic water content (Figure 7C,D). Serum amylase activity was significantly elevated after AP induction, and MO had no further effect on it (Figure 7F). However, AP did not induce any significant increase in pancreatic MPO activity (Figure 7E). MO had no additional effect on MPO activity during AP (Figure 7E). 

### 2.4. Buprenorphine Has No Effect on the Severity of LO-Induced AP

**Figure 8:**
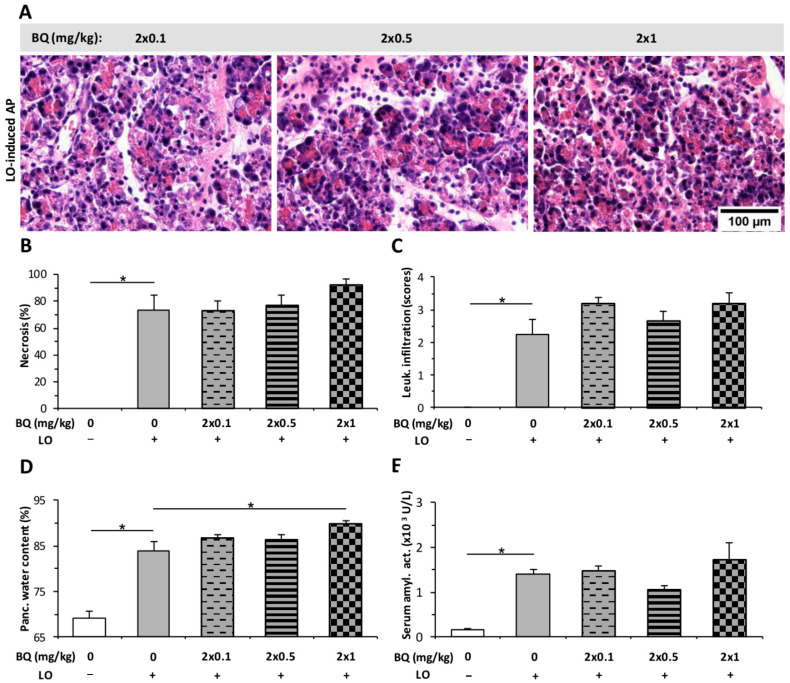
Intraperitoneal (i.p.) buprenorphine (BQ) treatment does not affect the severity of L-ornithine (LO)-induced acute pancreatitis (AP). Rats were treated with 2 × 0.1, 2 × 0.5, or 2 × 1 mg/kg BQ i.p., whereas i.p. injection with 3 g/kg LO (LO +) was used to induce AP. Control animals received physiological saline instead of LO (LO −) or BQ (0 mg/kg). Animals were sacrificed at 24 h after the first CER or physiological saline injection. (**A**) Representative histopathological images of pancreatic tissues of the treatment groups. Bar charts show the extent of pancreatic (**B**) necrosis, (**C**) leukocyte infiltration, (**D**) water content, and (**E**) serum amylase activity measurements. Values represent mean with standard error, *n* = 6. Two-way ANOVA was performed followed by the Holm–Sidak post hoc test. * *p* < 0.05.

The effect of BQ was tested by i.p. and i.t. administrations. BQ alone did not induce any changes in pancreatic tissues (Figure S1C). The tested i.p. doses (2 × 0.1; 2 × 0.5; 2 × 1 mg/kg) of BQ did not affect the LO-induced pancreatic necrosis, leukocyte infiltration, or serum amylase activity (Figure 8A–C,E). However, 2 × 1 mg/kg BQ slightly enhanced the pancreatic water content in AP (Figure 8D).

**Figure 9:**
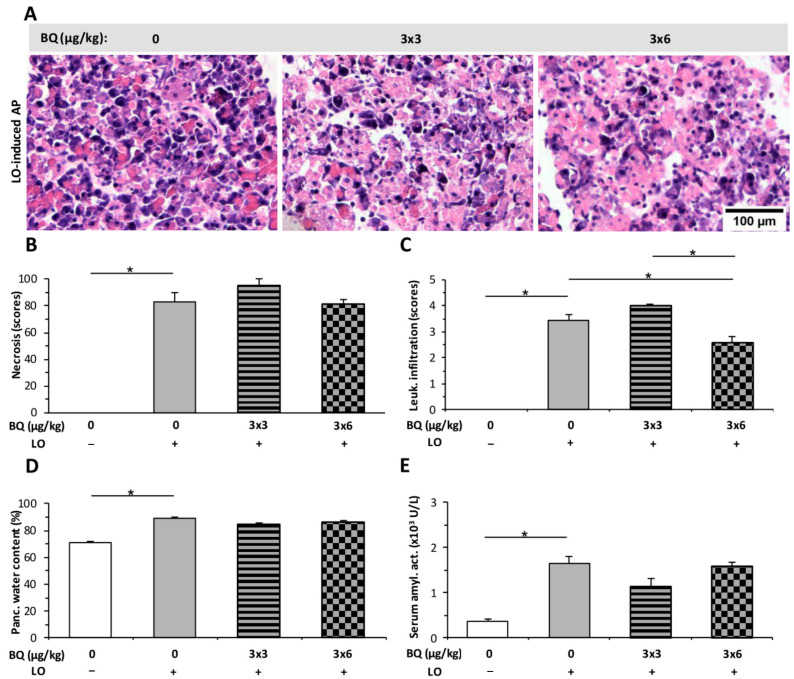
Intrathecal (i.t.) buprenorphine (BQ) treatment does not affect the severity of L-ornithine (LO)-induced acute pancreatitis (AP). Rats were treated with 3 × 3 or 3 × 6 mg/kg BQ i.t., whereas i.p. injection with 3 g/kg LO (LO +) was used to induce AP. Control animals received physiological saline instead of LO (LO −) or BQ (0 mg/kg). Animals were sacrificed at 24 h after the first CER or physiological saline injection. (**A**) Representative histopathological images of pancreatic tissues of the treatment groups. Bar charts show the extent of pancreatic (**B**) necrosis, (**C**) leukocyte infiltration, (**D**) water content, and (**E**) serum amylase activity measurements. Values represent mean with standard error, *n* = 6. ANOVA was performed followed by the Holm–Sidak post hoc test. * *p* < 0.05.

Intrathecal (i.t.) administration of BQ was also tested on rats during AP (Figure 9). The dose of 3 × 3 μg/kg BQ had no effect on any parameters of AP, while the 3 × 6 µg/kg dose significantly decreased the extent of leukocyte infiltration in AP (Figure 9C).

### 2.5. Pancreatic mu Opioid Receptor Expression Is Decreased in LO-Induced AP

**Figure 10:**
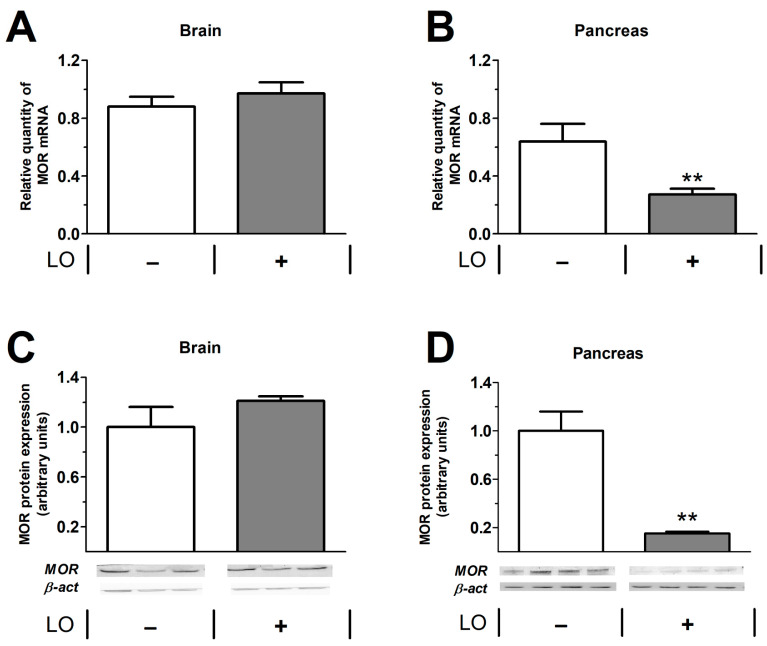
Expression of mu opioid receptor (MOR) in the brain and pancreas in control and LO-induced AP. Rats were treated with vehicle or 3 g/kg LO-HCl and were sacrificed at 24 h. MOR mRNA (**A**,**B**) and protein (**C**,**D**) expression levels were determined in the brain (**A**,**C**) and pancreas (**B**,**D**). In (**C**,**D**), the bar charts show the quantitative analysis of Western blot images. Values represent mean with standard error, *n* = 13–17 (RT-PCR), *n* = 3–4 (Western blot analysis). Student’s t test was performed, ** *p* < 0.01. Abbreviations: LO, L-ornithine-induced acute pancreatitis; β-act, β-actin; MOR, mu opioid receptor.

The mRNA and protein expression of mu opioid receptor (MOR) were investigated in the pancreas and brain (Figure 10). In the brain, MOR was detected in control animals, and AP did not influence the amount of MOR after 24 h (Figure 10A,C). In case of the pancreas, control animals also expressed MOR, but the induction of AP significantly reduced the presence of the receptor (Figure 10B,D).

### 2.6. Pancreatic and Brain mu Opioid Receptor Functions Are Reduced in AP

**Figure 11:**
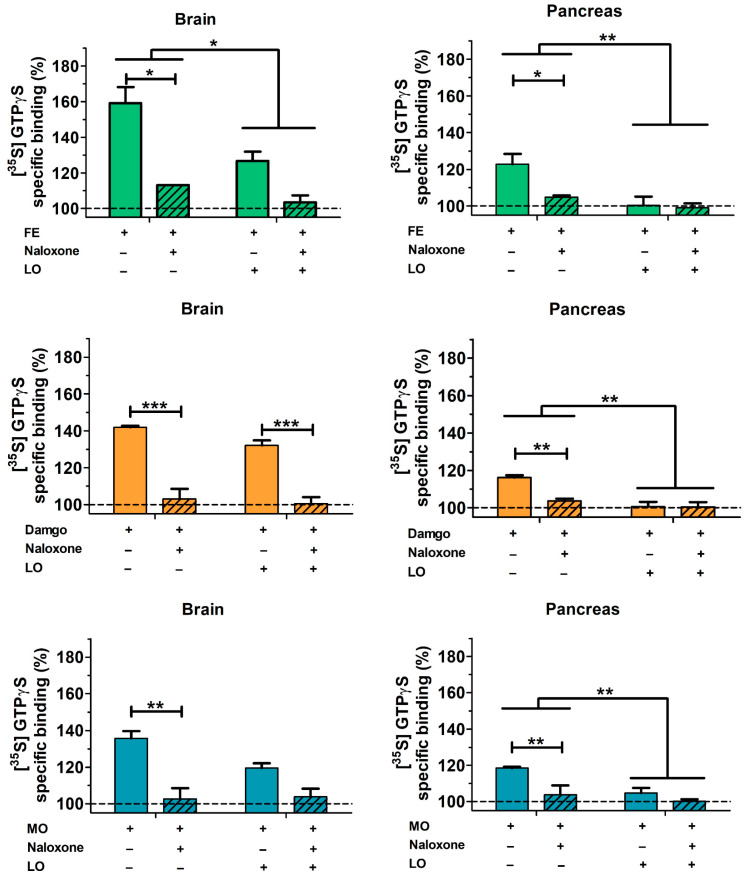
Stimulation of G-protein activation in rat brain and pancreas membrane homogenates. Tissue samples were derived from control and AP animals. Treatments of pancreatic homogenates were as follows: 10 µM FE; 10 µM DAMGO; 10 µM MO. Striped bars represent the combined treatment with mu receptor ligands (FE, MO, DAMGO) and equimolar naloxone. Values represent mean with standard error, *n* = 6. Two-way ANOVA was performed followed by Bonferroni post hoc test. * *p* < 0.05, ** *p* < 0.01; *** *p* < 0.001.

The functional activity of opioid receptors in pancreatic and brain-derived cell membrane homogenates were studied by receptor mediated in vitro G-protein stimulation (Figure 11). The G-protein activating effect of three well-known MOR agonists (FE, MO, and the highly selective synthetic opioid peptide Tyr-D-Ala-Gly-_(NMe)_Phe-Gly-ol—DAMGO) was measured at a concentration above the saturation level of the receptor (10 µM). The involvement of opioid receptors in G-protein activation was demonstrated by the inhibition with the well-known opioid receptor specific antagonist naloxone at equimolar concentration. In our experiments, brain and pancreatic preparations were investigated in animals with or without AP. All three tested agonists efficiently activated Gi/o proteins in guanosine-5′-[^35^S] thiophosphate ([^35^S]GTPγS) binding experiments. The level of pancreatic activation was lower than the corresponding values found in the brain samples (statistics were not performed in that comparison). In the pancreas, the rank order of efficacy of the activating agonist ligands was fentanyl > morphine ≅ DAMGO. The activation of G proteins was virtually eliminated in samples from AP compared to the control group (Figure 11).

## 3. Discussion

Opioids are commonly used for pain control in AP patients. It has been speculated that these analgesics (such as morphine) may affect AP progression. Therefore, we comprehensively investigated the effects of FE on the severity of experimental AP, and this research was further supplemented with the examination of the effects of MO and BQ. It is important to note that measurements were performed when the experimental AP reached its maximal severity.

I.p. FE pre-treatment significantly increased the severity of necrotizing AP induced by LO, but it had no effect on edematous AP evoked by CER. Interestingly, the clinically more relevant post-treatment with FE either decreased or had no effect on the various parameters of AP severity in different models. Wang and Chen [23] also tested the effect of FE on NaTc-induced AP. They administered FE i.v. 23–23.5 h after AP induction and sacrificed the animals 24 h after the induction of the disease. Surprisingly, FE exerted anti-inflammatory effects on the pancreas and AP-induced myocardial damage within that really short time (30–60 min). In clinical settings, Stevens et al. [24] showed that FE did not have any side effects compared to the placebo control group (intramuscular Demerol containing pethidine). Some studies draw attention to the importance of the administration site of FE, especially into the epidural site. The use of FE in epidural anesthesia partially restored the decrease in microcirculatory flow caused by AP and prevented the development of tissue necrosis and systemic complications [25,26]. 

MO pre- or post-treatment did not affect the severity of the disease in case of LO-induced necrotizing AP. Furthermore, the simultaneous administration of MO and CER had no remarkable effect on disease progression either, except for vacuolization, which was decreased by MO. In a recent study, Barlass et al. [21] also investigated MO in two necrotizing mouse AP models. They concluded that MO application delayed AP resolution and reduced intestinal motility, which increased the risk for bacterial translocation. MO also delayed macrophage migration and caused a persistence of inflammation. Their findings related to macrophages are in accordance with earlier studies showing mononuclear cell suppression and chemokine receptor transdeactivation after MO treatment [27,28]. Our study focused on the early-mid events of AP and showed no adverse effects of MO, while Barlass et al. [21] investigated the later effects of MO (at 48, 72, or 120 h). However, our results do not rule out the possibility of later side effects that were shown by Barlass et al. [21]. Marked differences in the results can be explained by species differences, the latter study used mice, while in the present study, rats were investigated. Moreover, one randomized clinical trial [29] and two related reviews [17,30] did not find any significant difference in the effects of MO vs. the non-opioid metamizole. It should be noted that a relatively low number of patients (eight per group) were included in this randomized clinical trial. Based on these observations, we conclude that MO does not affect the severity of the AP at the early-mid stage of the disease, but later side effects may appear according to literature data.

The partial opioid receptor agonist BQ did not cause any adverse effects during AP in i.p. pre-treatment; only tissue water content was increased by the highest dose. I.t injection of the smaller dose of BQ did not affect any other aspects of disease severity measured in our experiments. However, the higher dose significantly decreased immune cell infiltration. Based on this, i.t. administration could be more beneficial during experimental AP. Furthermore, we demonstrated first the effects of BQ on AP at the spinal level. Literature data showed that in an NaTc-induced AP rat model, i.v. BQ administration did not influence disease severity [31]. In a CER-AP model, subcutaneous 0.5 mg/kg BQ reduced the zymogen content and protein synthesis of acinar cells [32]. These results strengthen the beneficial effect of BQ during AP. 

Opioids exert their effects primarily through mu, kappa, or delta opioid receptors, which are expressed mainly by neuronal or immune cells. The effects can differ depending on their affinity or specificity to certain receptors. Publications showed that MO has immunosuppressant properties through full mu receptor agonism. MO treatment resulted in the inhibition of cytokine production, NK cell activity, cellular responses to mitogens, antibody production, cell growth, and decreased phagocytic activity [33,34]. FE is 80 times more potent than MO and is a highly selective full MOR agonist ligand [35]. Therefore, it can also suppress the immune system [19]. MO and FE can also cause a sphincter of Oddi spasm, which could further aggravate AP severity [36]. In contrast to MO and FE, BQ is a partial agonist of the mu receptor, while it is an antagonist of kappa and delta opioid receptors [19]. Therefore, BQ has a different pharmacological profile than the other opioids, and it does not inhibit NK cells, T cells, phagocytosis of macrophages, or cytokine production [19], and it has no morphine-like effect on the sphincter of Oddi [37]. These effects of opioids on cellular processes or on the sphincter of Oddi may explain the changes observed during AP in our experiments. Only FE pre-treatment resulted in increased AP severity. The early immunosuppression by FE may cause this adverse effect, while FE post-treatment was beneficial for AP outcome. However, the later consequences were not investigated by this work. For all clinically applied opioids, including FE, MO, or BQ, these effects should be considered and investigated in future studies. Moreover, the timing of opioid administration can be critical, especially in case of FE. 

We demonstrated MOR mRNA and protein expression in the control rat pancreas and brain. It is well known that the brain expresses large amounts of opioid receptors [38,39]; in case of the pancreas, other research groups have also shown MOR expression in rats [40], sheep [41], and humans [42]. Pancreatic islet cells express MOR [43], which influences glucose homeostasis and insulin secretion. There is no direct evidence on opioid receptor expression in exocrine pancreatic cells. However, it has been demonstrated that enkephalin and MO inhibit pancreatic bicarbonate and protein secretion during endogenous or exogenous stimulation (secretin or cholecystokinin-octapeptide) in dogs [44], which may indicate the presence of MOR in both acinar and ductal cells. Other opioid receptors (nociception/orphanin FQ and delta opioid receptors) also play a role in regulating exocrine pancreatic secretion [45]. Furthermore, pancreatic cholinergic neurons have opiate receptors as well [46].

The efficiency of G-protein stimulation by mu opioid agonists was markedly higher in the rat brain than in pancreatic preparations. Transmembrane signaling mediated by opioid agonists was almost completely eliminated in the pancreatic cell membrane preparations of AP animals at 24 h. This can be explained by the dramatic decrease in pancreatic MOR mRNA and protein expression. In case of brain tissue, no reduction in MOR protein and mRNA levels could be observed. At 24 h, pancreatic tissue necrosis is extensive, which can contribute to the reduction of different receptors such as MOR, while there is no tissue necrosis in the brain; therefore, MOR expression remained unaltered. To the best of our knowledge, we demonstrated for the first time that AP reduced the function of opioid receptors not only in the pancreas but also in the brain. Notably, other groups have shown that mu opioid receptor expression is upregulated in hind paw or intestinal inflammatory animal models [47,48]. However, tissue acidification induced by injury or inflammation impaired MOR signaling [49]. Since the extent of AP severity is influenced by FE acting via opioid receptors (predominantly on MOR), we wanted to check their expression in the pancreas and brain and their functional activity in cell membrane fractions prepared from both tissues. The expression of MOR in the brain was unchanged in response to AP, whereas its functional activity was decreased during FE stimulation. This means that AP may affect MOR activity independently of changes in protein expression. The increase in serum pro-inflammatory cytokine (interleukin 1β) concentration has been shown to reduce central opioid neurotransmitter function [50]. Furthermore, there is a crosstalk between chemokines and opioid receptors, since certain chemokines (e.g., CCR2, CCR5, CCR7, CXCR4) can desensitize opioid receptors [34]. The most prominent symptom of AP is pain. During the disease, endogenous opioids (such as enkephalins, endorphins, and dynorphins) are released [51]. These substances may cause MOR desensitization [52,53], which could also contribute to the observed reduction in MOR activity. Moreover, high amounts of MOR are expressed in the spinal cord, which modulates pain sensation via the descending pain pathway system [54]. It is known that chronic pancreatitis causes chronic pain, which will result in epigenetic modulations of pain-related genes [55]. The latter is mediated by increased histone deacetylase 2 activity during chronic pancreatitis in the spinal cord. Consequently, there will be a reduction of MOR expression within some weeks. AP lasts for a shorter period, but due to the persistent pain, MOR expression can also be affected in the spinal cord. Further studies could investigate MOR not just in the pancreas and brain but also in the spinal cord. Overall, the mechanisms by which AP affects opioid receptor activity is partly unknown, but we must infer a very likely interaction between the biochemical processes of opioid ligand binding and G-protein-mediated transmembrane signaling and organ inflammation.

In the clinical setting, there are no guidelines or recommendations suggesting which is the best opioid to use in AP. However, the application of effective and strong analgesics is necessary in the treatment of this disease. In light of the results discussed above, post-treatments (e.g., FE, MO) do not increase disease severity, but some of the opioids (e.g., MO) may affect the resolution of AP. Therefore, the latter may not be the best treatment option in this severe disease. Our results showed that FE post- and BQ pre-treatments have promising effects besides pain relief; therefore, the use of these opioids could also be beneficial for AP severity. Overall, this research contributes to a better understanding of the opioid effect in AP and can help design further clinical trials that will be necessary to select the most appropriate opiate to treat this potentially lethal disease.

Although pre-treatment with analgesics in AP is clinically less relevant, rectal administration of nonsteroidal anti-inflammatory drugs (NSAIDs, e.g., indomethacin or diclofenac) is indicated for endoscopic retrograde cholangiopancreatography (ERCP) [56]. These agents reduce the development of post-ERCP-related AP. In this case, the use of opiates could be also tested. 

The present study has limitations as well. The long-term consequences of opiates on AP were not investigated as it was performed by Barlass et al. [21]. Furthermore, the above-mentioned and beneficial epidural administration route [25,26] was not investigated by our group.

In conclusion, we showed for the first time that AP reduced the transmembrane signaling of mu opioid receptors in both the pancreas and the brain. We demonstrated that FE post-treatment improved, while FE pre-treatment exacerbated disease severity in necrotizing AP. However, FE did not affect the outcome of edematous AP. MO administration had minimal effects in both pre- and post-treatments including cellular vacuolization, pancreatic water content, and leukocyte infiltration. I.t. administration of BQ showed slight benefit over i.p. injection. FE post-treatment proved to be beneficial in AP. Finally, our results suggest that type, dosing, administration route, and timing of opioid treatment can determine the effects on AP outcome. Clinical studies are needed to determine which opioid(s) is the best in AP.

## 4. Materials and Methods

### 4.1. Animals

Female Wistar rats weighing 200–250 g were used for experiments. The animals were kept at a constant room temperature of 24 °C with a 12 h light–dark cycle and were allowed free access to water and standard laboratory chow (Biofarm, Zagyvaszántó, Hungary). 

### 4.2. Materials

All chemicals were purchased from Sigma-Aldrich (Budapest, Hungary) unless indicated otherwise. 

### 4.3. In Vivo Experiments: Acute Pancreatitis Induction, Opiate Treatments, and Tissue Collection

**Figure 12:**
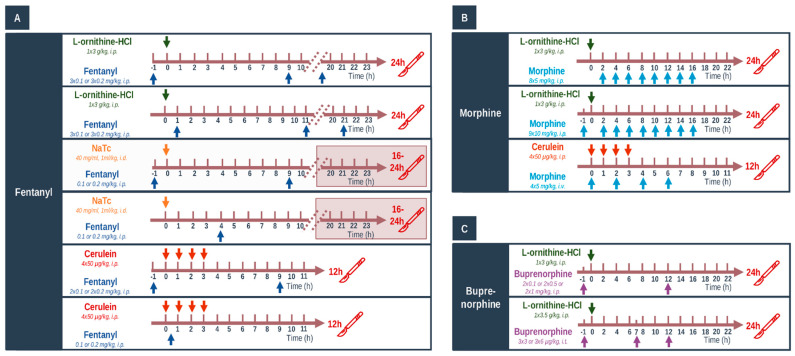
(**A**–**C**) Schematic view of experimental setup. Treatment arrangements for acute pancreatitis (AP) induction and opioid administration in Wistar rats. Arrows above or below the timeline show the injections. Control animals were injected with physiological saline. Abbreviations: i.p.—intraperitoneal; i.t.—intrathecal; i.v.—intravenous.

Three different models of AP were applied (Figure 12). Necrotizing AP was induced by (a) single i.p. injection of 3 g/kg L-ornithine-HCl (LO, 30%, pH = 7.4); (b) intra-ductal administration of 1 mL/kg Na-taurocholate solution (NaTc; 40 mg/mL) as described previously [9,22]. Edematous AP was induced by hourly i.p. injections of 20 µg/kg cerulein (CER, 50 µg/mL) four times. Briefly, in case of NaTc-induced AP, abdominal surgery was performed on anesthetized rats (with 70 mg/kg ketamine and 14 mg/kg xylazine i.p.—purchased from CP-Pharma-Handelsgesellschaft MBH (Burgdorf, Germany)). Then, a cannula was placed into the pancreatic duct, and the biliary duct was transiently occluded via a microvessel clip. The NaTc solution was injected at a speed of 50 µL/min. At the end of the procedure, rats were placed on a heating pad for 40 min or until they woke up. Thereafter, rats were placed back into their cages for 16–24 h. Control groups were given physiological saline (0.9% NaCl) solution instead of LO/CER/NaTc, respectively. Animals were sacrificed at 24 h in the LO-induced experimental pancreatitis model, between 16 and 24 h in case of the NaTc model, and at 12 h in case of the CER model. In case of NaTc-induced AP, rats were extensively monitored, and when body temperature decreased below 30 °C, they were humanely sacrificed by deep anesthesia induced by 85 mg/kg i.p. pentobarbital injection (Bimeda MTC, Cambridge, ON, Canada).

FE was administered i.p. at doses of 0.1 and 0.2 mg/kg based on the literature data [57]. Different timing arrangements were applied for FE in various AP models; repeated injections were performed when the analgesic effect of FE was decreased (this was determined in preliminary experiments or by literature data). In addition, FE was used as pre- or post-treatment. In the pre-treatment groups, the first FE injection was given 1 h prior to the induction of AP, and it was repeated every 11 h in LO- and every 10 h in NaTc- or CER-induced AP, respectively (Figure 12A). In preliminary experiments, FE pre-treatment was also tested in NaTc-induced AP, but the condition of animals was critical; therefore, humane termination was performed, and these investigations were stopped. In the post-treatment setup, animals received the first FE injection 1 h after AP induction in case of the LO model or 0.5 h after AP induction in case of the CER model. Since FE depresses respiration [58], it could not be administered within 3 h after surgery; therefore, FE was injected 4 h after the beginning of surgery in case of the NaTc model of AP (Figure 12A).

In the post-treatment setup, 5 mg/kg MO was administered i.p. 8 times every 2 h in case of the LO model (Figure 12B). The dose and timing of MO was chosen based on literature data; repeated injections were performed when the analgesic effect of MO was decreased [59]. During pre-treatment, 10 mg/kg MO was injected i.p. 9 times every 2 h (Figure 12B). When AP was induced by CER, 4 × 5 mg/kg dose of MO was used i.v. every 2 h, and analgesia started simultaneously with AP induction (Figure 12B). Animals were sacrificed 24 or 12 h after AP induction with LO or CER, respectively.

BQ has prolonged analgesic effects, and its recommended dosing intervals are between 8 and 12 h [60]. Instead of testing BQ in different AP models, it was administered via two routes: i.p and intrathecally (i.t., Figure 12C). For i.t. administration, rats were anesthetized with a mixture of ketamine hydrochloride and xylazine (72 and 8 mg/kg i.p, respectively). An i.t. catheter (PE-10 tubing Intramedic, Clay Adams; Becton Dickinson; Parsippany, NJ, USA; I.D. 0.28 mm; O.D. 0.61 mm) was inserted via the cistern magna and passed 8.5 cm caudally into the subarachnoid space [61], which served to place the catheter tip between vertebrae Th12 and L2 vertebrae, corresponding to the spinal segments that innervate the hind paws [62]. After surgery, animals were injected by gentamycin (10 mg/kg, subcutaneously) to prevent infection and were housed individually. Rats exhibiting postoperative neurologic deficits, or those ones that did not show paralysis of one of the hindpaws after the administration of 100 µg lidocaine were excluded (about 10%) [62]. The drugs were applied at least after 4 days of recovery. I.p. injections of 0.1, 0.5, and 1 mg/kg BQ were given 1 h before and 12 h after the beginning of AP induction. I.t. injections of 3 and 6 µg/kg BQ were administered 1 h before AP induction and were repeated at 7 and 12 h after AP induction with LO. BQ was injected over 120 s in a volume of 10 µL, which was followed by a 10 µL flush of physiological saline. These BQ doses are in accordance with literature data [63,64]. 

At the end of experiments/treatments, deep anesthesia was induced by 85 mg/kg i.p. pentobarbital injection. Blood was collected through cardiac puncture; then, the pancreas was rapidly removed. Pancreata were cleaned from fat and lymph nodes on ice and then cut into pieces. Two parts of the pancreatic tissue were immediately frozen in liquid nitrogen and stored at −80 °C until biochemical assays or dry–wet weight measurements were performed. The third part of the pancreas was fixed in 8% neutral formaldehyde solution for histological analysis. In case of the NaTc model, pancreata were stored only for histological analysis due to the heterogeneity of AP induction. Blood samples were centrifuged at 2500 RCF for 15 min at 4 °C, and the sera were stored at −20 °C until use. Brains were also rapidly collected from rats, and the whole tissues were used for [^35^S]GTPγS functional binding assay, whereas the cortex was used for PCR and Western blots. Brain samples were stored at −80 °C until further processing.

### 4.4. Laboratory Measurements

Serum amylase activity was measured on a Fluorostar Optima plate reader (BMG Labtech, Ortenberg, Germany) with a colorimetric kinetic method using a commercial kit purchased from Diagnosticum Zrt. (Budapest, Hungary). To evaluate the pancreatic water content, the wet weight (WW) of the pancreata was measured; then, the tissues were dried for 24 h at 100 °C, and the dry weight (DW) was also measured. The wet/dry weight ratio was calculated as follows: [(WW−DW)/WW] × 100. Pancreatic myeloperoxidase (MPO) activity is a hallmark of leukocytic infiltration and was measured according to Kuebler et al. [65]. MPO activities were normalized to total protein content as measured by the Lowry method [66]. To determine the extent of inflammatory response in the pancreata, we measured interleukin (IL)-1β levels by a commercial ELISA kit from R&D Systems (Minneapolis, MN, USA), as described by the manufacturer.

### 4.5. Histological Examination 

Formalin-fixed pancreatic tissues were sectioned to 3 µm. These sections were prepared and stained with hematoxylin and eosin and were analyzed and scored by two independent experts blinded to the experimental protocol. Five different random areas were observed and scored per section per researcher. Edema was scored between 0 and 3 points (0: none; 1: patchy interlobular; 2: diffuse interlobular; 3: diffuse interlobular and intra-acinar), leukocytic infiltration between 0 and 4 points (0: none; 1: diffuse/mild; 2: diffuse/moderate; 3: diffuse/severe; 4: diffuse/very severe), vacuolization between 0 and 3 points (0: none; 1: mild; 2: moderate; 3: severe); the percentage of acinar cell damage was also evaluated.

### 4.6. Total RNA Preparation from Tissue

A small piece of pancreas or brain cortex was placed on ice in 1 mL of TRIzol reagent in a 13 mL centrifuge tube and was homogenized immediately with IKA Ultra Turrax (Type: TP18/10; Janke and Kunkel IKA, Staufen im Breisgau, Germany). Then, the tissue homogenate was instantly placed on liquid nitrogen and stored at −80 °C until use (for a maximum of 1 or 2 days). Total RNA purification was performed in three steps. In the first step, phase separation was performed by adding 200 μL of chloroform to the samples and shaking vigorously for 15 min, allowing to stand, and then centrifuging at 12,000 *g* for 15 min at 4 °C. From the resulting three phases, the top aqueous phase was aspirated into an empty Eppendorf tube, and 500 μL of isopropanol was added. Then, this was vortexed and allowed to stand for a few minutes, and after that, it was centrifuged at 12,000 *g* for 10 min at 4 °C. RNA precipitated in the Eppendorf tubes. The supernatant was removed, and 1 mL of 75% alcohol was added. It was vortexed and centrifuged at 7500 *g* for 5 min at 4 °C. After removal of the supernatant, the excess ethanol was evaporated briefly, and then, the RNA was redissolved in 70 μL of RNAse-free water. RNA was stored at −80 °C until further use.

RNA concentration was measured using a NanoDrop instrument from Thermo Fisher Scientific. We considered the optimal ranges for RNA to be A260/A280: 1.9–2.1 and A260/A230: 1.8–2.5. RNA integrity was examined after agarose gel electrophoresis.

### 4.7. Real-Time Quantitative Reverse Transcription-PCR (RT-PCR)

Reverse transcription and amplification of the PCR products were performed by using the TaqMan RNA-to-CT-Step One Kit (Thermo Fisher Scientific, Budapest, Hungary) and an ABI StepOne Real-Time cycler (Applied Biosystems, Thermo Fisher Scientific). Reverse-transcriptase PCR amplifications were performed as follows: at 48 °C for 15 min and at 95 °C for 10 min, followed by 40 cycles at 95 °C for 15 s and at 60 °C for 1 min. The generation of specific PCR products was confirmed by melting curve analysis. The following primers were used: assay ID Rn01430371_m1 for Oprm1 and Rn00667869_m1 for β-actin as endogenous control (Thermo Fisher Scientific). Each sample was run in triplicates. The fluorescence intensities of the probes were plotted against PCR cycle number. The amplification cycle displaying the first significant increase in the fluorescence signal was defined as the threshold cycle (Ct). Relative quantity of MOR mRNA expression was calculated by using the 2^−^^ΔΔCt^ method. 

### 4.8. Western Blot Analysis

Pancreatic and brain tissues were homogenized using a Micro-Dismembrator (Sartorius AG, Göttingen, Germany) and centrifuged at 5000 g for 15 min at 4 °C in RIPA Lysis and Extraction Buffer (Thermo Fisher Scientific) with a protease and phosphatase inhibitor cocktail (10 mM Na-HEPES, 1 µM MgCl_2_, 10 mM KCl, 1 mM DL-dithiothreitol, 5 mM iodoacetamide, 4 mM benzamidine-HCl, 1 mM phenylmethyl sulfonylfluoride). Total protein amounts from supernatant were determined with spectrophotometry (BioSpec-nano, Shimadzu, Kyoto, Japan).

Then, 25 µg of protein per well was subjected to electrophoresis on 4–12% NuPAGE Bis-Tris Gel in XCell SureLock Mini-Cell Units (Thermo Fisher Scientific). Proteins were transferred from gels to nitrocellulose membranes, using the iBlot Gel Transfer System (Thermo Fisher Scientific). Antibody binding was detected with the WesternBreeze Chromogenic Western blot immunodetection kit (Thermo Fisher Scientific). The blots were incubated on a shaker with OPRM1 (1:200, cat. no.: AOR-011, Alomone Labs, Jerusalem, Israel) and β-actin (cat. no.: bs-0061R, 1:200, Bioss Antibody, Woburn, MA, USA) polyclonal antibodies in the blocking buffer. Images were captured with the EDAS290 imaging system (Kodak Ltd., Rochester, NY, USA), and the optical density of each immunoreactive band was determined with Kodak 1D Images analysis software. Optical densities were calculated as arbitrary units after local area background subtraction. MOR expression was corrected for β-actin levels. Values were normalized to control groups.

### 4.9. Preparation of Brain and Pancreas Samples for Binding Assays

Frozen rat brain and pancreas samples from LO or physiological saline-treated animals were prepared for membrane preparation according to Szűcs et al. [67]. Briefly, tissue samples were homogenized in 30 volumes (*v*/*w*) of ice-cold 50 mM Tris-HCl pH 7.4 buffer (containing 4 mM benzamidine hydrochloride hydrate, 1 mM phenylmethyl sulfonylfluoride (Serva Electrophoresis GmbH, Heidelberg, Germany), 5 mM iodoacetamide, and 1 mM DL-dithiothreitol (Fluka Honeywell Research Chemicals, Charlotte, NC, USA)) with a Teflon-glass Braun homogenizer operating at 1500 rpm. The homogenate was centrifuged at 40,000 rcm for 20 min at 4 °C, after which the pellet was taken up in the original volume of Tris-HCl buffer. The homogenate was incubated at 37 °C for 30 min in a shaking water-bath. Then, centrifugation was repeated as described before. The final pellet was suspended in 5 volumes of TEM buffer (50 mM Tris-HCl, 1 mM EGTA, 5 mM MgCl_2_, pH 7.4) and stored at −80 °C.

### 4.10. [^35^S]GTPγS Functional Binding Assay

The functional [^35^S]GTPγS binding experiments were performed as previously described [68]. Briefly, the membrane proteins (≈10 µg/mL) were incubated at 30 °C for 60 min with [^35^S]GTPγS (20 MBq/0.05 cm^3^; 0.05 nM; Perkin Elmer, Boston, MA, USA) and with 10 µM FE, DAMGO (Bachem Holding AG, Bubendorf, Switzerland) or MO in Tris-EGTA buffer (containing 30 µM GDP, 1 mM EGTA, 5 mM MgCl_2_, 100 mM NaCl, and 50 mM Tris-HCl, pH 7.4) in a final volume of 1 mL/reaction tube. The non-selective opioid receptor antagonist naloxone (Endo Laboratories DuPont de Nemours, Wilmington, DE, USA) was used to detect receptor specificity. Non-specific binding was determined with 10 µM unlabeled GTPγS and subtracted from total binding. Basal activity (was defined as 100%) indicates constitutive G-protein activity level in the absence of any stimulating ligand. Bound and free [^35^S]GTPγS were separated by vacuum (Brandel M24R Cell Harvester) filtration through Whatman GF/B glass fiber filters washed three times with 5 mL of ice-cold 50 mM Tris-HCl (pH 7.4) buffer. The results were performed in triplicates and repeated at least three times.

### 4.11. Statistical Analysis

The sufficient animal number per group was estimated by power analysis before each experiment, using the G*Power (3.1.9.2., Heinrich-Heine-Universität Düsseldorf, Germany) software [69] and setting the effect size to 0.8. Data are presented as means ± SEM. Experiments were evaluated by Student’s *t*-test or by one- or two-way ANOVA followed by Holm–Sidak or Bonferroni post hoc tests (SPSS, IBM, Armonk, NY, USA). *p* < 0.05 was accepted as statistically significant.

## Data Availability

The datasets generated during and/or analyzed during the current study are available from the corresponding author on reasonable request.

## Supplementary Materials

The following are available online at https://www.mdpi.com/article/10.3390/ijms23031192/s1.
