# Supplementary material for: Fentanyl but Not Morphine or Buprenorphine Improves the Severity of Necrotizing Acute Pancreatitis in Rats
Source: Int J Mol Sci. 2022 Jan 21;23(3):1192. doi: 10.3390/ijms23031192 (PMC8835441; doi:10.3390/ijms23031192)
Supplement: Supplementary file 1 [file ijms-23-01192-s001.zip › ijms-1336079-supplementary.pdf]

Fentanyl but not morphine or buprenorphine improves the severity of necrotizing acute pancreatitis in rats

Emese R. Bálint[1], Gabriella Fűr[1], Balázs Kui[2], Zsolt Balla[1], Eszter S. Kormányos[1], Erik M. Orján[1], Brigitta Tóth[1], Gyöngyi Horváth[3], Edina Szűcs[4], Sándor Benyhe[4], Eszter Ducza[5], Petra Pallagi[2], József Maléth[2], Viktória Venglovecz[6], Péter Hegyi[7,8], Lóránd Kiss[1*], Zoltán Rakonczay Jr.[1*]

1 Department of Pathophysiology, University of Szeged, Szeged, Hungary
2 Department of Medicine, University of Szeged, Szeged, Hungary
3 Department of Physiology, University of Szeged, Szeged, Hungary
4 Institute of Biochemistry, Biological Research Center, Szeged, Hungary
5 Department of Pharmacodynamics and Biopharmacy, Faculty of Pharmacy, University of Szeged, Szeged, Hungary
6 Department of Pharmacology and Pharmacotherapy, University of Szeged, Szeged, Hungary
7 Institute for Translational Medicine, University of Pécs, Pécs, Hungary
8 MTA-SZTE Momentum Translational Gastroenterology Research Group, University of Szeged, Szeged, Hungary

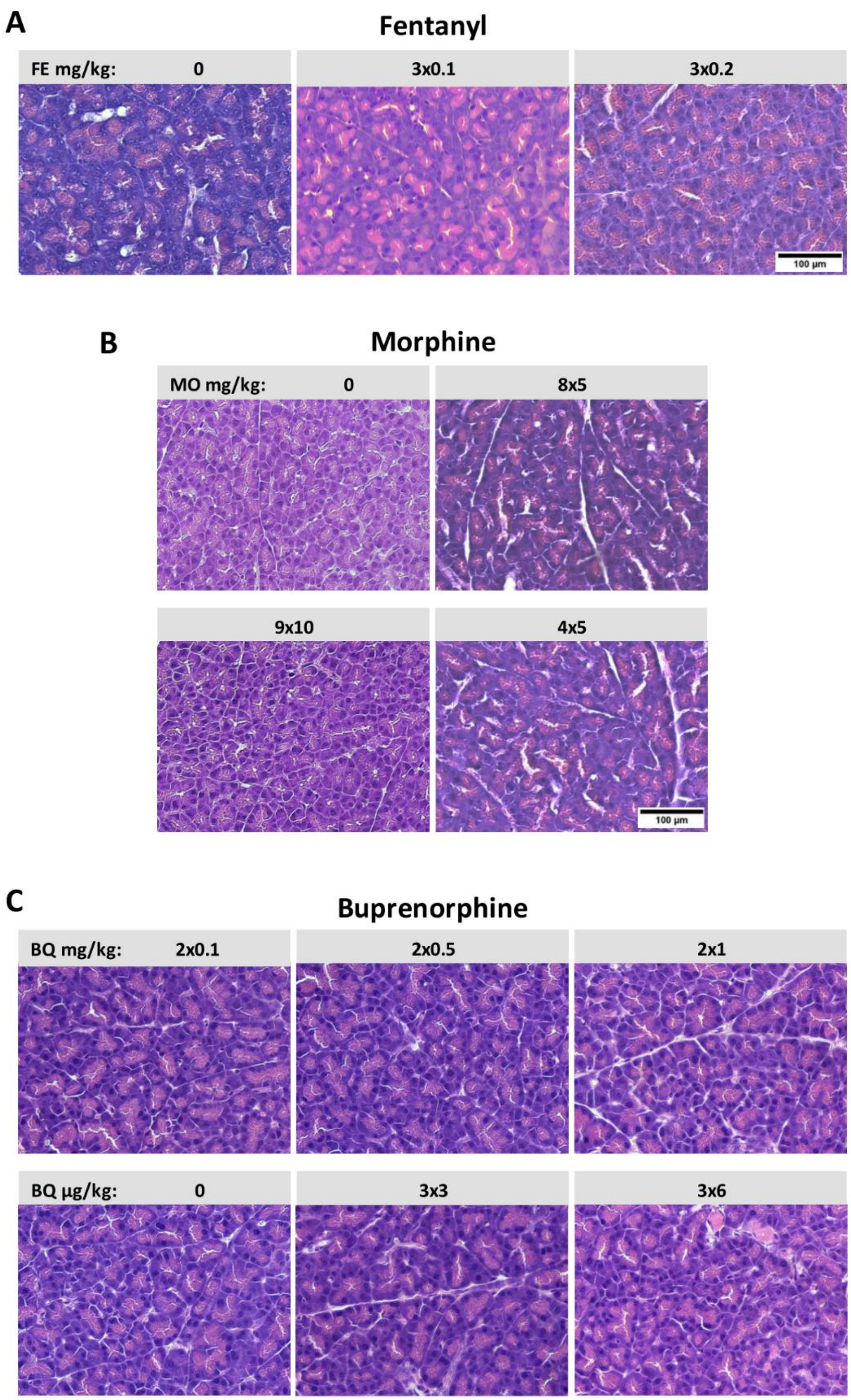

Figure S1. Representative histopathological images of pancreatic tissues of the control groups in cases of (**A**) fentanyl (FE), (**B**) morphine (MO) and (**C**) buprenorphine (BQ) treatments. Doses of opioids used are provided above each image.
